# Neuropsychiatric symptoms in a patient with Dyke–Davidoff–Masson syndrome and systemic lupus erythematosus: a case report

**DOI:** 10.1186/s13256-019-2039-2

**Published:** 2019-04-29

**Authors:** José Sordia-Ramírez, Adrián Infante-Valenzuela, Iván de Jesús Hernández-Galarza, Antonio Costilla-Esquivel

**Affiliations:** 1 Department of Psychiatry, University Hospital “Dr. José E. González”, Autonomous University of Nuevo León, Monterrey, Nuevo León México; 2 Neurology Service, Internal Medicine Department, University Hospital “Dr. José E. González”, Autonomous University of Nuevo León, Monterrey, Nuevo León México; 3 Rheumatology Service, Internal Medicine Department, University Hospital “Dr. José E. González”, Autonomous University of Nuevo León, Monterrey, Nuevo León México

**Keywords:** Neuropsychiatric symptoms, Dyke–Davidoff–Masson syndrome, Systemic lupus erythematosus, Depression, Antiphospholipid syndrome

## Abstract

**Background:**

Dyke–Davidoff–Masson syndrome is an uncommon constellation of radiological and clinical findings. Few reports describe co-occurring psychiatric manifestations. Systemic lupus erythematosus is a systemic disease with vascular, neurologic, and psychiatric involvement. To the best of our knowledge, no case reports have been made associating these entities.

**Case presentation:**

We present the case of a 21-year-old Mexican mestizo woman with a history of systemic lupus erythematosus diagnosed at 4 years of age, who developed focal impaired awareness seizures when she was 8-years old, which became treatment-resistant at age 15. Two years prior to our evaluation, she developed deep vein thrombosis; clinical and laboratory criteria were met to diagnose secondary antiphospholipid syndrome. After being treated with anticonvulsants, glucocorticoids, and immunosuppressants with only a partial response, she developed a severe major depressive episode 1 year prior to our assessment, including two suicide attempts. She was referred to the out-patient clinic of our department for evaluation; intellectual disability, depressive symptoms, and behavioral symptoms were documented. Imaging studies revealed structural abnormalities in the left cerebral hemisphere: cortical atrophy, enlargement of sulci and cisternal spaces, and hyperpneumatization of the frontal sinus. Treatment with an antidepressant was initiated and maintained for 1 year, added to anticonvulsants and immunosuppressants. Depressive and behavioral symptoms diminished and no suicidal ideation has been noted at follow-up.

**Conclusions:**

Dyke–Davidoff–Masson syndrome was diagnosed, accompanied by clinical symptoms previously reported as epilepsy and intellectual disability. This case report illustrates the complexity of syndrome presentation in an adult female, constituting a diagnostic and therapeutic challenge. This constellation of symptoms and structural brain abnormalities should be kept in mind in patients with neuropsychiatric manifestations and systemic diseases with central nervous system involvement, especially when diagnosed at a young age.

## Background

Dyke–Davidoff–Masson syndrome (DDMS) was first described in 1933 [1] as a rare radiological set of features that depend on age at diagnosis and underlying cause. The brain imaging diagnostic findings are: cerebral hemiatrophy; enlargement of ipsilateral sulci, ventricles, and cisternal spaces; compensatory skull thickening; and ipsilateral hyperpneumatization of sinuses [2]. Clinical features such as hemiplegia/hemiparesis, facial asymmetry, treatment-resistant epilepsy, and intellectual disability have been described too, although, their presentation is variable [3, 4]. Psychiatric disorders reported in association with DDMS encompass childhood-onset schizophrenia, schizoaffective disorder, treatment-resistant psychosis, and bipolar disorder in a manic episode [5–8].

Systemic lupus erythematosus (SLE) is a chronic, multisystem autoimmune disorder that typically affects young women, involves vascular manifestations in up to 50% of cases, and frequently includes neurological and psychiatric symptoms [9]. Antiphospholipid syndrome (APS) is an autoimmune disorder where thrombosis is the main pathophysiological feature, affecting veins and arteries; it causes obstetric complications, with high comorbidity alongside SLE [10]. We present the case of a patient with DDMS, SLE, and APS exhibiting affective and behavioral disturbances. To the best of our knowledge, no cases in which these conditions co-occur have been reported.

## Case presentation

Our patient is a 21-year-old Mexican mestizo woman with a family history of SLE (her father had the diagnosis), who at age 4 developed malar rash, fever, anemia, fatigue, and malaise. She was hospitalized, received a SLE diagnosis, and began taking corticosteroids and immunosuppressive agents, with constant disease flares throughout her early years. At 6 years of age, she developed an episode of septic monoarthritis in her right knee, requiring surgical drainage and antibiotics. Speech and attention problems were noted at this age, along with irritability, apathy, and lack of concentration at school. At 8 years of age, she began experiencing seizures that consisted of a visceral aura (butterflies in the stomach, as referred by the patient), fixed gaze, altered consciousness, oral and buccal automatisms, somnolence, and amnesia of the event at the postictal phase. These seizures occurred once a week approximately and were diagnosed as focal impaired awareness seizures, originating from the left medial temporal lobe. Anticonvulsants provided good control of the seizures until age 15 when these seizures became treatment-resistant.

At age 19 she was received in our hospital with a 3-week evolution symptomatology of generalized fatigue, localized pain, hyperthermia, pruritus, and hyperemia of her right lower extremity. Deep vein thrombosis was diagnosed with Doppler ultrasound, from the right popliteal vein through the right femoral vein, and laboratory tests revealed hemoglobin (Hb) of 4.83 g/dL, mean corpuscular volume (MCV) of 54.6 fL, mean corpuscular Hb (MCH) of 15.1 pg, and reticulocyte count of 5.6%. A lupus anticoagulant test was positive and she was diagnosed as having secondary APS and microcytic hypochromic anemia, requiring anticoagulants and blood transfusions for her treatment. She was prescribed hydroxychloroquine, prednisone, azathioprine, warfarin, calcium, and vitamin D supplements at discharge. The neurological treatment of her seizures consisted of levetiracetam, lamotrigine, magnesium valproate, and phenobarbital.

She then developed a severe major depressive episode as defined by the *Diagnostic and Statistical Manual of Mental Disorders*, Fifth Edition (DSM-5) [11]; characterized by anhedonia, abulia, mixed insomnia, depressed mood, and suicidal thoughts; behavioral disturbances were noted including mainly impulsiveness and irritability. During this period, she performed two suicide attempts, the second and more severe occurred 5 months after APS diagnosis, when she ingested lamotrigine and magnesium valproate in an unknown quantity. She was admitted to the emergency room of our hospital with altered consciousness, nausea, dizziness, and weakness. She presented seizures 36 hours after stabilization with the same semiology as mentioned above; despite treatment, the seizures worsened and developed into status epilepticus, and she had to be transferred to our intensive care unit for mechanical intubation and sedation. Electroencephalogram studies showed interictal activity of focal seizures arising from the left frontotemporal region, with secondary generalization. At discharge, rheumatologic and neurologic follow-up was scheduled, and she was referred to the psychiatry department. She had not had a psychiatric or cognitive evaluation before.

During assessment at our clinic, she denied a history of alcohol or drug abuse, but presented with labile affect and emotional dysregulation, symptoms like abulia, mixed insomnia, impulsiveness, suicidal thoughts, and irritability were noted as the most disabling. Psychotic or manic symptoms were not present at the time of evaluation, so these diagnostic spectrums were ruled out. At physical examination, she had normal vital signs: blood pressure 90/60 mmHg, heart rate 87 beats per minute, respiratory rate 14 per minute, and temperature 36.8 °C. She had a normal height and weight of 1.46 m and 47.5 kg respectively, with a body mass index (BMI) of 22.30. A neurologic examination showed normal motor and sensitive findings; a cranial nerve examination was normal too. She did not present paresis of any extremities. Isolated hyperreflexia of lower extremities was noted (+++), glabellar and right palmomental reflex were present, and there were no other signs or symptoms of upper/lower motor neuron disease. Seizure symptomatology was still active, but with a better response than previously in her treatment course (one seizure every 2/3 weeks, approximately).

**Fig. 1:**
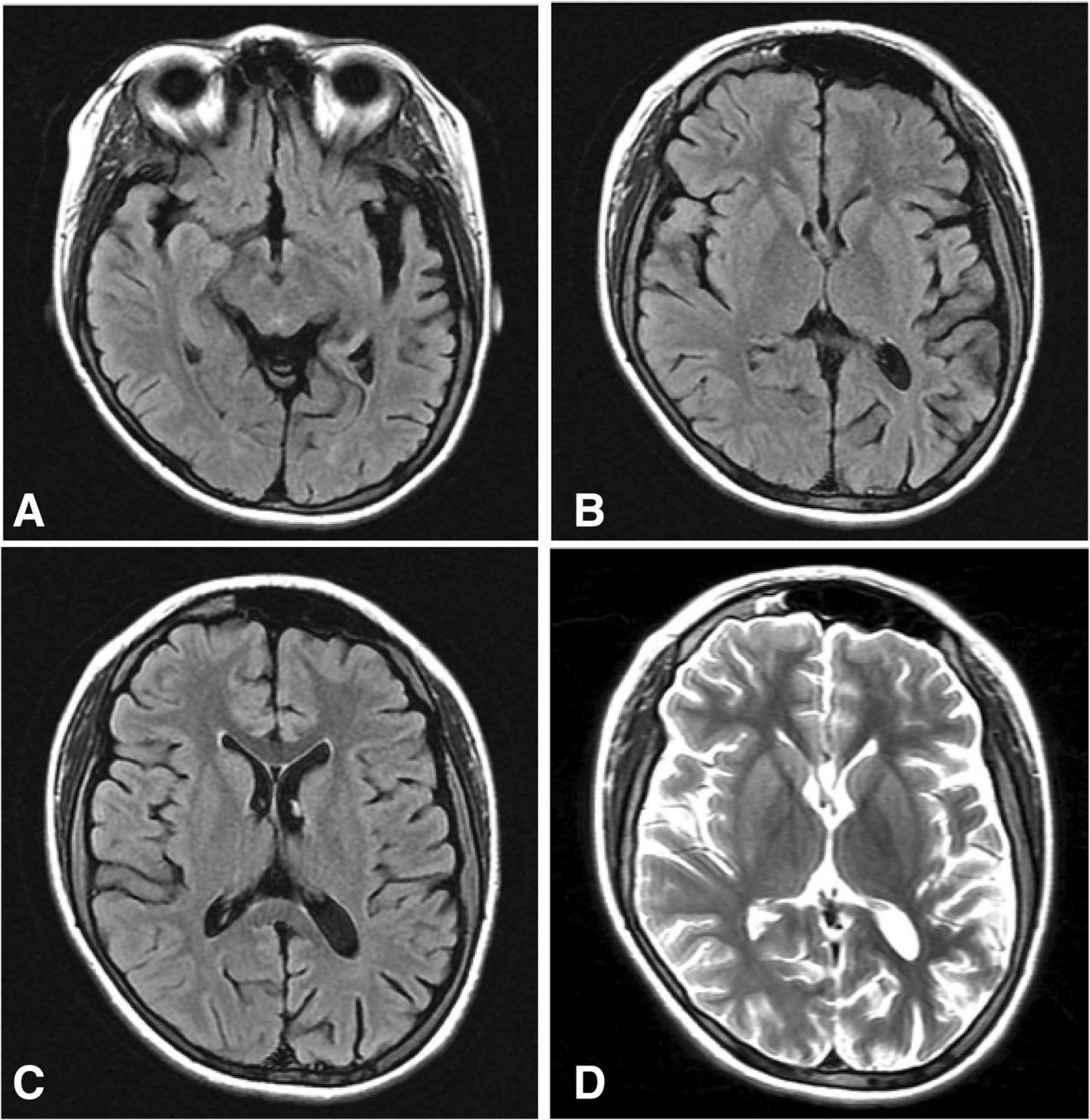
Brain magnetic resonance imaging. **a**–**c** Axial T2-weighted fluid-attenuated inversion recovery images showing left cerebral hemisphere cortical atrophy, with ipsilateral skull thickening and hyperpneumatization of the left frontal sinus, along with enlargement of the atrium, frontal, temporal, and posterior horns of the left lateral ventricle. Widening of sulci, cisternal spaces, and subarachnoid space is also noted. **d** Axial T2 weighted image showing the same description mentioned above

**Fig. 2:**
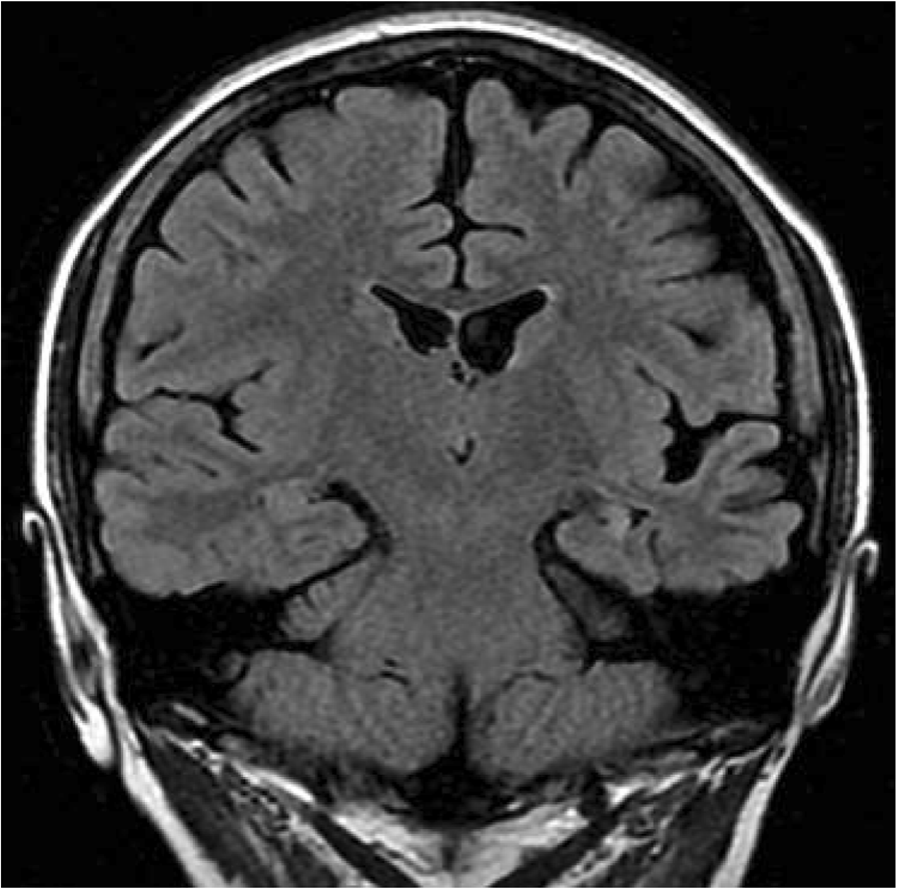
Brain magnetic resonance imaging. Coronal T2-weighted fluid-attenuated inversion recovery image showing left hippocampal hypotrophy, widening of sulci and subarachnoid space in left parietal and temporal lobes, and dilation of the left lateral ventricle

Magnetic resonance imaging (MRI) was performed and revealed neuroanatomical structural changes most noticeable in the left cerebral hemisphere, including generalized cortical atrophy with skull thickening and ipsilateral widening of sulci, ventricles, and cisternal spaces; hypotrophy of the hippocampus and medial temporal lobe structures was also noted (Figs. 1a–d and 2). She was cognitively evaluated, scoring 15/30 points in the Montreal Cognitive Assessment (MoCA) test and 20/30 points in the Mini-Mental State Examination (MMSE). In the Wechsler Adult Intelligence Scale-IV (WAIS-IV) she received a total intelligence quotient of 65 (extremely low scoring range), which together with her academic history and current coping abilities indicated a moderate intellectual disability according to DSM-5 criteria [11]. In subscale analysis, she had a Processing Speed Index (PSI) of 77, Verbal Comprehension Index (VCI) of 70, and the most affected areas of the test were Perceptual Reasoning Index (PRI) with a score of 66, and Working Memory Index (WMI) with a score of 55, both of them in an extremely low score range (Table 1).

**Table 1:** Wechsler Adult Intelligence Scale-IV results

	Coefficient	Classification	Confidence interval (95%)	Percentile range
Verbal Comprehension Index	70	Borderline	65–78	2
Perceptual Reasoning Index	66	Extremely low	61–74	1
Working Memory Index	55	Extremely low	51–64	0.1
Processing Speed Index	77	Borderline	71–88	6
Total intelligence quotient	65	Extremely low	61–71	1

Because of residual depressive symptoms at the time of evaluation, she was prescribed citalopram 20 mg per day, and behavioral interventions to improve conduct symptoms. She had mild improvement in her psychiatric symptomatology with these measures, but depressive symptoms did not evolve into a full recurrent major depressive episode, irritability and impulsiveness were controlled, and her adherence to treatment improved in all her psychiatric, neurologic, and rheumatologic treatments and follow-up visits. The last pharmacological regimen included citalopram, lamotrigine, and topiramate (for depressive symptoms and seizure control) and hydroxychloroquine, azathioprine, warfarin, prednisone, and vitamin D (for SLE and APS), with partial treatment response to these medications as shown by persistent hematologic manifestations. Despite the absence of gastrointestinal bleeding as evidenced by repeated negative fecal immunochemical tests results, she has suffered several episodes of microcytic hypochromic anemia.

## Discussion

Our patient had DDMS, SLE, and APS along with depressed mood, suicidal ideation, and suicidal attempts, after which she developed status epilepticus. While psychiatric symptoms in patients with DDMS have been previously reported, we were unable to locate a published description of the co-occurrence of this syndrome with severe neuropsychiatric manifestations of SLE, including suicidality.

Patients with DDMS are most commonly male in gender; our patient is female, but she did display a left cerebral hemisphere involvement predominantly, as has been reported [3, 12]. Congenital causes of DDMS include malformations, birth trauma, vascular injury, and intracranial hemorrhage. Tumors, trauma, hemorrhage, ischemia, and infectious diseases, like the one she had as a young girl, have been associated with the development of acquired DDMS [2]. Neurologic symptoms like treatment-resistant epilepsy, status epilepticus, and intellectual disability also have been reported previously in patients with DDMS [3, 13, 14]. Cerebral atrophy stems from a diminished production of neurotrophic factors, which in turn arises from an insult during brain development [15].

It is unclear if the neuropsychiatric dysfunction observed in our patient is due to one or all of her ailments. The time course suggests microvascular and autoimmune involvement as a possible contributor to DDMS. Patients with SLE have been shown to display blood–brain barrier dysfunction, as inferred from results of serum and cerebrospinal fluid (CSF) analysis showing increased levels of immunoglobulins, proinflammatory cytokines, and albumin; furthermore, specific antibodies have been related to psychiatric symptoms in these patients, such as anti-ribosomal P protein antibodies, associated with lupus psychosis and severe depression [9, 16]. Vascular damage is also involved in the presentation of central nervous system (CNS) signs and symptoms in these patients: multi-focal microinfarcts, small-vessel vasculopathy mediated through deposition of immune complexes and complement activation, and microhemorrhages are among the most commonly reported [10, 16]. Patients with SLE and positive antiphospholipid antibodies develop thrombotic events in up to 50% of cases; although thrombosis can occur in any vessel, up to 90% of episodes are deep vein thromboses in the legs, as our patient had [10]. Anemia is a common manifestation in patients with inflammatory rheumatologic conditions; complement and/or antibody-mediated hemolysis should be considered a persistent hematologic manifestation of lupus in our patient, as is seen in up to 10% of these patients; moreover, a combination of iron deficiency anemia and anemia of chronic disease has been described in patients with SLE, with glucocorticoid treatment and nonsteroidal anti-inflammatory drugs (NSAIDs) often contributing to its origin [17]. Cognitive dysfunction is common in patients with SLE, with particular impact in visual and verbal memory, attention, and executive functions, independent of serological or systemic disease activity. Seizures are seen in up to 25% of patients with SLE [16]. Cortical hypotrophy in the left medial temporal lobe was recognized as the origin of focal seizures in our patient.

Although there are previous reports of association with autoimmune diseases like large vessel granulomatous vasculitis [18], to the best of our knowledge this is the first case that reports DDMS alongside SLE and APS. The unusual presentation of SLE in our patient, being diagnosed at 4 years of age, with vascular and neurological complications at such a young age is important. Similar to other cases of DDMS associated with psychiatric symptoms, in our patient DDMS was identified after psychiatric presentation of clinical symptoms [5–8].

Drug prescription and dosage should be carefully coordinated between physicians involved in complex cases like this. Even though full-fledged psychiatric conditions are not common presentations for persons with DDMS, they can result in chaotic scenarios due to the antipsychotic drugs seizure threshold-lowering effects in a context of difficult-to-treat seizures. Pharmacokinetic processes of absorption, distribution, metabolism, and excretion may be altered in a patient with SLE, APS, and polypharmacy. Protein-binding properties, cytochrome P450 (CYP) isoenzymes activity, and renal excretion are the main mechanisms involved in drug–drug interactions. Highly protein-bound drugs like warfarin and magnesium valproate were used in this patient, small changes in unbound fractions of these drugs could lead to serious untoward effects, and special consideration should be made in a patient at greater risk of protein synthesis dysfunction due to chronic multisystem diseases like this one. Our patient received phenobarbital, a potent inducer of CYP2C9 and CYP3A4, which could have had an impact on warfarin’s anticoagulant therapeutic effects; it is also known to interfere with lamotrigine and levetiracetam in this way. Magnesium valproate is known to be a potent inhibitor of lamotrigine’s hepatic glucuronidation, and it may increase serum levels of phenobarbital via this mechanism too [19]. All of these medications were used by our patient at the same time prior to her first suicide attempt, which leads to a reasonable presumption of possible psychotropic side effects due to interactions between these drugs throughout her treatment.

Citalopram was chosen over other antidepressants due to its small interaction profile with narrow therapeutic index agents like warfarin; it minimally inhibits CYP2D6, and CYP3A4 [20]. Although hepatic metabolism and renal excretion abnormalities were not documented as liver and renal function tests were normal, periodic evaluations should be made since the increased risk of damage to these organs due to SLE, APS, and/or drug-induced is considerable.

Psychiatric adverse drug reactions should also be considered in this patient; drugs prescribed throughout her treatment (glucocorticoids, levetiracetam, topiramate, valproate, and chloroquine) are known to be associated with psychiatric symptoms like depression, psychosis, cognitive dysfunction, behavioral changes, suicidal ideation, and sleep disturbances. New onset or worsening of neuropsychiatric symptoms after treatment changes for SLE, APS, and epilepsy may have had an impact on this patient’s psychiatric clinical presentation [21]. A high suspicion index for medical etiology when assessing any mental status changes in this patient is necessary.

## Conclusions

Patients with this constellation of symptoms should be evaluated and treated in a multisystemic approach to identify structural, cognitive, psychiatric, and neurologic complications, particularly in patients with autoimmune diseases that involve CNS manifestations like SLE and the side effects of medications used to treat these conditions. Cognitive rehabilitation, seizure control, control of mood and thought disorders, management of suicide risk, and autoimmune disease control are the main priorities to be accomplished when managing these patients for improvement in quality of life and prognosis.

## Abbreviations

APS: Antiphospholipid syndrome
BMI: Body mass index
CNS: Central nervous system
CSF: Cerebrospinal fluid
CYP: Cytochrome P450
DDMS: Dyke–Davidoff–Masson syndrome
DSM-5: *Diagnostic and Statistical Manual of Mental Disorders*, Fifth Edition
Hb: Hemoglobin
MCH: Mean corpuscular hemoglobin
MCV: Mean corpuscular volume
MMSE: Mini-Mental State Examination
MoCA: Montreal Cognitive Assessment
MRI: Magnetic resonance imaging
NSAIDs: Nonsteroidal anti-inflammatory drugs
PRI: Perceptual Reasoning Index
PSI: Processing Speed Index
SLE: Systemic lupus erythematosus
VCI: Verbal Comprehension Index
WAIS-IV: Wechsler Adult Intelligence Scale-IV
WMI: Working Memory Index
